# Cardiorenal Syndrome: From Hemodynamic and Humoral Pathophysiology to New Frontiers in Modulation and Enabling Therapy—Part 2

**DOI:** 10.3390/biomedicines14102257

**Published:** 2026-10-06

**Authors:** Alfredo Mauriello, Valeria Cetoretta, Carmen Del Giudice, Adriana Correra, Anna Chiara Maratea, Renato Gioia, Biagio Liccardo, Giovanni Esposito, Vincenzo Russo, Antonello D’Andrea

**Affiliations:** 1 S.C. Cardiology, Institute National Cancer, IRCCS, Fondazione “G. Pascale”, Via M. Semmola 52, 80131 Naples, Italy; alfredo.mauriello93@libero.it; 2 Cardiology and Arrhythmology Clinic, Marche Polytechnic University, University Hospital “Ospedali Riuniti”, Via Conca 71, 60126 Ancona, Italy; vcetoretta@gmail.com; 3 Cardiology Unit, Boscotrecase Hospital, ASL NA3Sud, Via Lenza, 3, 80042 Boscotrecase, Italy; carmen.delgiu93@gmail.com; 4 Cardiology Department, Ospedali Riuniti University Hospital, Viale Pinto 1, 71122 Foggia, Italy; adrianacorrera@gmail.com; 5 Department of Cardiovascular Disease, ASL Napoli 1 Centro, Via Comunale del Principe, 13a, 80145 Napoli, Italy; annachiara.maratea@gmail.com; 6 Cardiology and Intensive Care Unit, Department of Cardiology, Umberto I Hospital, Via A. de Nicola 1, 84014 Nocera Inferiore, Italy; renatogioia@aslsalerno.it; 7 Cardiology Unit, Department of Medical and Translational Sciences, Monaldi Hospital, University of Campania “Luigi Vanvitelli”, Via L. Bianchi snc, 80131 Naples, Italy; liccardob@gmail.com (B.L.); vincenzo.russo@unicampania.it (V.R.); 8 Cardiology Unit, Department of Advanced Biomedical Sciences, University of Naples Federico II, Via S. Pansini 5, 80131 Naples, Italy; giovanni.esposito2@unina.it

**Keywords:** cardiorenal syndrome, biomarker, SGLT2i, finerenone, GLP-1 agonists

## Abstract

The therapeutic landscape of cardiorenal syndrome (CRS) has evolved significantly, transitioning from historical cornerstones like angiotensin-converting enzyme (ACE) inhibitors and angiotensin receptor blockers (ARBs) to modern neurohormonal modulation with angiotensin receptor–neprilysin inhibitors (ARNIs). This second part of this narrative review analyzes the pivotal pharmacological roles of sodium–glucose cotransporter-2 (SGLT2) inhibitors, glucagon-like peptide-1 (GLP-1) receptor agonists, and finerenone, which target the hemodynamic, metabolic, and inflammatory–fibrotic axes, respectively, alongside the essential utility of novel potassium binders. Ultimately, the management of CRS is shifting toward an integrated cardiovascular–renal–metabolic paradigm, where the future lies in a personalized, biomarker-guided “quadruple cardiorenal therapy” that moves beyond a single-organ focus to optimize patient outcomes.

## 1. Introduction

For a long time, cardiorenal syndrome (CRS) was defined as the hemodynamic and humoral feedback loops between the heart and kidneys. Recently, however, this perspective has expanded into the broader cardiovascular–kidney–metabolic (CKM) framework. Rather than looking at the heart and kidneys in isolation, the CKM model recognizes that metabolic dysfunction, such as insulin resistance, obesity, and systemic inflammation, acts as the upstream catalyst for multisystem disease. From this viewpoint, CRS is best understood as a late-stage, specific phenotype of heart–kidney failure, while the CKM continuum captures the entire disease trajectory, from early metabolic risk to multiorgan decline.

Following Part 1 of this review, which broke down the pathophysiological mechanisms of bidirectional heart–kidney crosstalk, Part 2 explores how these insights translate into clinical practice. We examine how newer drug classes do more than just correct the hemodynamic imbalances of CRS; they actively modify the inflammatory and metabolic drivers underlying the broader CKM continuum.

Although structured as a narrative review, we applied a rigorous literature selection process. We searched PubMed, Scopus, and Web of Science for articles published between January 2010 and September 2026. Using keywords such as “cardiorenal syndrome,” “cardiovascular-kidney-metabolic,” “SGLT2 inhibitors,” “GLP-1 receptor agonists,” “finerenone,” and “potassium binders,” we prioritized landmark randomized controlled trials, major cardiovascular and renal outcome studies, and recent international guidelines. This approach ensures a focused and up-to-date overview of contemporary cardiorenal–metabolic management.

## 2. RAAS Inhibition and ARNI: From Historical Backbone to Modern Neurohormonal Modulation

RAAS inhibition represents the historical backbone of cardiorenal protection [1,2]. Its clinical value was first established in HF and post-MI left ventricular dysfunction. In heart failure with reduced ejection fraction (HFrEF), enalapril reduced all-cause mortality in Studies of Left Ventricular Dysfunction (SOLVD)-Treatment, with a 16% relative risk reduction, and reduced HF hospitalizations, establishing ACE inhibition as foundational therapy in systolic HF [3]. In the post-infarction setting, captopril, ramipril, and trandolapril in Survival and Ventricular Enlargement (SAVE), Acute Infarction Ramipril Efficacy (AIRE), and Trandolapril Cardiac Evaluation (TRACE) consistently reduced mortality and HF progression among patients with left ventricular dysfunction or clinical HF after MI [4,5,6]. The benefit was then extended beyond overt HF: in the Heart Outcomes Prevention Evaluation (HOPE) trial, ramipril reduced the composite of cardiovascular death, MI, or stroke in high-risk vascular patients, including many with diabetes mellitus type 2 (T2D) [7], while the EUropean trial on Reduction Of cardiac events with Perindopril in patients with stable coronary Artery disease (EUROPA) showed that perindopril reduced cardiovascular events in stable coronary artery disease without apparent HF [8]. The clinical development of ACE inhibitors and angiotensin receptor blockers, from landmark heart failure and post-MI trials to studies establishing nephroprotection in diabetic and non-diabetic chronic kidney disease (CKD), is summarized in Table 1.

In parallel, RAAS blockade became the first disease-modifying strategy for proteinuric CKD. The nephroprotective rationale was based on reduction of intraglomerular pressure and albuminuria, effects that extend beyond systemic blood pressure lowering. In diabetic kidney disease, losartan in Reduction of Endpoints in NIDDM with the Angiotensin II Antagonist Losartan (RENAAL) and irbesartan in Irbesartan Diabetic Nephropathy Trial (IDNT) reduced hard renal outcomes, including doubling of serum creatinine and progression to end-stage kidney disease [13,14], while Irbesartan in patients with T2D and Microalbuminuria (IRMA-2) showed that irbesartan delayed progression from microalbuminuria to overt nephropathy. Similarly, ramipril in Ramipril in non-diabetic renal failure (REIN) slowed renal function decline in proteinuric non-diabetic CKD [11,12]. These trials established albuminuria both as a therapeutic target and a marker of residual renal risk.

However, despite these foundational benefits, substantial residual cardiovascular and renal risk persisted. This created the rationale for angiotensin receptor–neprilysin inhibition (ARNI), which combines blockade of angiotensin II signaling with enhancement of endogenous natriuretic peptide activity. In Prospective Comparison of ARNI with angiotensin-converting enzyme inhibitors (ACEI) to Determine Impact on Global Mortality and Morbidity in Heart Failure (PARADIGM-HF), sacubitril/valsartan was superior to enalapril in HFrEF, reducing cardiovascular death or HF hospitalization by 20% (HR 0.80, 95% CI 0.73–0.87; *p* < 0.001), with parallel reductions in cardiovascular and all-cause mortality. In Prospective Comparison of ARNI with ARB Global Outcomes in HFpEF (PARAGON-HF), the same strategy narrowly missed statistical significance in heart failure with preserved ejection fraction (HFpEF) for the composite of total HF hospitalizations and cardiovascular death (rate ratio 0.87, 95% CI 0.75–1.01; *p* = 0.06) [15], but subgroup and pooled analyses suggested greater benefit in patients with lower ejection fraction within the preserved range. Prospective ARNI vs. ACE Inhibitor Trial to Determine Superiority in Reducing Heart Failure Events After MI (PARADISE-MI) did not significantly reduce cardiovascular death, HF hospitalization, or outpatient HF after acute MI compared with ramipril (HR 0.90, 95% CI 0.78–1.04; *p* = 0.17) [16], while Prospective Comparison of ARNI With ARB Given Following Stabilization In Decompensated HFpEF (PARAGLIDE-HF) showed greater NT-proBNP reduction with sacubitril/valsartan than valsartan in recent worsening HF with mildly reduced (HFmrEF) or HFpEF [17].

Beyond their established cardiovascular benefits, ARNIs have also demonstrated favorable effects on renal function and cardiorenal physiology. By combining RAAS blockade with neprilysin inhibition, sacubitril/valsartan enhances endogenous natriuretic peptide activity, promoting natriuresis, vasodilation, and attenuation of intraglomerular hypertension. In both PARADIGM-HF and PARAGON-HF, treatment with sacubitril/valsartan was associated with a slower rate of eGFR decline compared with enalapril or valsartan, respectively, despite a modest increase in albuminuria. This apparent dissociation suggests that albuminuria changes under neprilysin inhibition may not necessarily reflect worsening kidney injury but rather alterations in glomerular hemodynamics. Furthermore, sustained reductions in natriuretic peptide concentrations observed across ARNI trials indicate effective unloading of the cardiorenal axis and may contribute to long-term preservation of both cardiac and renal function. These findings support the concept that ARNIs provide integrated cardiorenal protection extending beyond conventional neurohormonal blockade. Overall, RAAS inhibitors remain the historical foundation of cardiorenal protection, whereas ARNIs represent the evolution of neurohormonal modulation toward a more integrated cardiorenal strategy, combining cardiovascular benefits with favorable effects on renal function and cardiorenal hemodynamics.

The pivotal trials of ARNI across the cardiorenal continuum are summarized in Table 2.

## 3. SGLT2 Inhibitors: From Glucose-Lowering Agents to Cardiorenal Disease-Modifying Therapies

The introduction of sodium–glucose cotransporter-2 (SGLT2) inhibitors has profoundly reshaped the management of patients across the spectrum of cardiovascular and kidney disease. Initially developed as antihyperglycemic agents, these drugs rapidly demonstrated benefits that extended far beyond glycemic control, leading to a paradigm shift in the treatment of T2D, CKD, and HF. The first evidence of cardiovascular protection emerged from the EMPA-REG OUTCOME trial, in which empagliflozin significantly reduced major adverse cardiovascular events (MACE) in patients with T2D and established cardiovascular disease (HR 0.86, 95% CI 0.74–0.99; *p* = 0.04) [19]. Remarkably, this benefit was driven by a substantial reduction in cardiovascular mortality (HR 0.62, 95% CI 0.49–0.77; *p* < 0.001) accompanied by reductions in all-cause mortality and hospitalization for heart failure, with separation of event curves occurring within the first months after randomization. Subsequent cardiovascular outcome trials confirmed that the benefits of SGLT2 inhibition represent a class effect. In the CANVAS Program, canagliflozin reduced the risk of MACE by 14% and demonstrated early signals of nephroprotection through attenuation of albuminuria progression and preservation of renal function [20]. Similarly, DECLARE–TIMI 58, which enrolled a broader population, including a large proportion of primary prevention patients, did not significantly reduce MACE but significantly lowered the composite of cardiovascular death or hospitalization for heart failure (HR 0.83, 95% CI 0.73–0.95; *p* = 0.005) [21], mainly driven by a reduction in HF hospitalization, while simultaneously improving renal outcomes. The VERTIS-CV trial with ertugliflozin further confirmed cardiovascular safety and provided additional evidence supporting favorable effects on heart failure and renal outcomes despite neutral effects on MACE [22]. A major milestone in the evolution of the field was the demonstration that SGLT2 inhibitors exert direct nephroprotective effects. In CREDENCE, the first dedicated renal outcome trial, canagliflozin reduced the risk of end-stage kidney disease (ESKD), doubling of serum creatinine, or renal/cardiovascular death by 30% (HR 0.70, 95% CI 0.59–0.82; *p* = 0.00001) in patients with diabetic kidney disease already receiving optimized renin–angiotensin system blockade [23]. Importantly, the trial also showed significant reductions in hospitalization for heart failure and MACE, highlighting the inseparable nature of cardiovascular and renal protection.

The concept of cardiorenal protection independent of diabetes was subsequently es-tablished by DAPA-CKD and EMPA-KIDNEY. In DAPA-CKD, dapagliflozin reduced the composite of sustained ≥50% eGFR decline, ESKD, or renal/cardiovascular death by 39% (HR 0.61, 95% CI 0.51–0.72; *p* < 0.001) while also reducing cardiovascular death or heart failure hospitalization (HR 0.71, 95% CI 0.55–0.92; *p* = 0.009) and all-cause mortality (HR 0.69, 95% CI 0.53–0.88; *p* = 0.004). Notably, these benefits were similar in patients with and without diabetes [24]. EMPA-KIDNEY expanded these findings to an even broader CKD population, including patients with lower levels of albuminuria and non-diabetic kidney disease, demonstrating a significant reduction in kidney disease progression or cardio-vascular death (HR 0.72, 95% CI 0.64–0.82; *p* < 0.001) [25]. Collectively, these studies es-tablished SGLT2 inhibitors as foundational nephroprotective therapies alongside renin–angiotensin system inhibitors.

Parallel advances have occurred in heart failure. DAPA-HF and EMPEROR-Reduced demonstrated that dapagliflozin and empagliflozin reduce worsening HF and cardiovas-cular death in patients with HFrEF regardless of diabetic status. In DAPA-HF, dapagli-flozin reduced the primary composite endpoint of worsening HF or cardiovascular death by 26% (HR 0.74, 95% CI 0.65–0.85; *p* < 0.001) [26], while EMPEROR-Reduced showed a similar 25% reduction (HR 0.75, 95% CI 0.65–0.86; *p* < 0.001) together with a slower de-cline in eGFR [27]. The consistency of these results strongly suggested that the cardiovas-cular benefits of SGLT2 inhibitors were largely independent of glycemic control and po-tentially mediated through hemodynamic, metabolic, renal, and myocardial mechanisms. The therapeutic spectrum was subsequently expanded to HF with mildly reduced and preserved ejection fraction. EMPEROR-Preserved became the first positive trial in HFpEF, showing a significant reduction in cardiovascular death or HF hospitalization (HR 0.79, 95% CI 0.69–0.90; *p* < 0.001) [28]. These findings were confirmed by DELIVER, in which dapagliflozin reduced worsening HF or cardiovascular death in patients with LVEF > 40% (HR 0.82, 95% CI 0.73–0.92; *p* < 0.001) [29]. Together, these studies established SGLT2 in-hibitors as the first pharmacological class capable of improving outcomes across the entire left ventricular ejection fraction spectrum.

Additional evidence came from sotagliflozin, a dual SGLT1/SGLT2 inhibitor. In SCORED, patients with T2D and CKD experienced a significant reduction in cardiovas-cular death, HF hospitalization, or urgent HF visits (HR 0.74, 95% CI 0.63–0.88; *p* < 0.001) [30], whereas SOLOIST-WHF demonstrated that initiation shortly after hospitalization for worsening HF reduced recurrent HF events and cardiovascular death (HR 0.67, 95% CI 0.52–0.85; *p* < 0.001) [31].

The clinical development of SGLT2 inhibitors, from glucose-lowering therapies to disease-modifying agents with proven cardiovascular and renal benefits across a broad range of patient populations, is summarized in Table 3.

The evidence from cardiovascular outcome trials, dedicated renal studies, and heart failure trials consistently indicates that SGLT2 inhibitors are no longer simply glucose-lowering agents but rather foundational cardiorenal therapies. Their ability to simultaneously reduce heart failure events, slow CKD progression, preserve renal function, and improve survival across diabetic and non-diabetic populations has positioned them at the center of contemporary cardiorenal medicine. The remarkable consistency of benefits across diverse clinical settings strongly supports the concept of a shared cardiorenal protective mechanism, involving restoration of tubuloglomerular feedback, reduction in intraglomerular hypertension, optimization of myocardial energetics, attenuation of neurohormonal activation, and improvement of systemic congestion.

## 4. GLP-1 Receptor Agonists and Cardiorenal Protection

Glucagon-like peptide-1 receptor agonists (GLP-1 RAs) have evolved from glucose-lowering agents to therapies capable of modifying cardiovascular and renal risk in patients with type 2 diabetes mellitus (T2DM). In contrast to SGLT2 inhibitors, whose benefits are predominantly driven by reductions in heart failure and chronic kidney disease (CKD) progression, GLP-1 RAs exert their protective effects mainly through improvements in metabolic control, weight reduction, blood pressure lowering, attenuation of systemic inflammation, and anti-atherosclerotic mechanisms. Consequently, their clinical benefits have been most consistently observed in the prevention of atherosclerotic cardiovascular events.

Cardiovascular outcome trials (CVOTs) initially demonstrated heterogeneity within the class. ELIXA and EXSCEL established the cardiovascular safety of lixisenatide and exenatide, respectively, but failed to demonstrate superiority over placebo for major ad-verse cardiovascular events (MACE) [33,34]. In contrast, several human GLP-1-based ago-nists showed significant cardiovascular benefit. In the LEADER trial, liraglutide reduced the risk of cardiovascular death, nonfatal myocardial infarction, or nonfatal stroke by 13% (HR 0.87, 95% CI 0.78–0.97; *p* = 0.01), with a notable 22% reduction in cardiovascular mortality (HR 0.78, 95% CI 0.66–0.93; *p* = 0.007) [35]. Similarly, SUSTAIN-6 demonstrated a 26% reduction in MACE with semaglutide (HR 0.74, 95% CI 0.58–0.95), driven predom-inantly by a reduction in nonfatal stroke [36]. The cardiovascular benefits of the class were subsequently confirmed across broader populations. In REWIND, dulaglutide signifi-cantly reduced MACE in patients with and without established cardiovascular disease (HR 0.88, 95% CI 0.79–0.99; *p* = 0.026) [37], extending the evidence base beyond secondary prevention settings. Likewise, HARMONY Outcomes showed that albiglutide reduced MACE by 22% (HR 0.78, 95% CI 0.68–0.90; *p* = 0.0006) [38], with a particularly pro-nounced reduction in myocardial infarction. More recently, AMPLITUDE-O demonstrated that efpeglenatide reduced MACE by 27% (HR 0.73, 95% CI 0.58–0.92; *p* = 0.007) [39], con-firming that cardiovascular protection extends across structurally diverse members of the GLP-1 RA class, including exendin-based compounds. The cardiovascular benefits of GLP-1 receptor agonists were further extended beyond diabetes by the SELECT trial, in which semaglutide 2.4 mg weekly reduced major adverse cardiovascular events by 20% in overweight or obese patients with established cardiovascular disease but without diabetes (HR 0.80, 95% CI 0.72–0.90; *p* < 0.001) [40]. These findings suggest that the cardioprotec-tive effects of GLP-1 receptor agonists are not solely mediated by glycemic control and support their role as cardiovascular risk-modifying agents across the broader cardiomet-abolic spectrum. Evidence supporting renal protection has evolved substantially over time. Early CVOTs suggested that GLP-1 RAs primarily reduced albuminuria and delayed the onset of macroalbuminuria. In LEADER and SUSTAIN-6, favorable renal outcomes were largely driven by reductions in new or worsening nephropathy, particularly persistent macroalbuminuria. However, these studies were not specifically designed to evaluate kidney outcomes and included relatively few hard renal events. AMPLITUDE-O provided stronger evidence, demonstrating a 32% reduction in a composite renal outcome, includ-ing decline in kidney function or macroalbuminuria (HR 0.68, 95% CI 0.57–0.79; *p* < 0.001) [39]. The definitive evidence for nephroprotection emerged with the FLOW trial, the first dedicated renal outcome study of a GLP-1 receptor agonist. In patients with T2DM and CKD, semaglutide reduced the risk of major kidney disease events, defined as kidney fail-ure, a sustained ≥50% decline in estimated glomerular filtration rate (eGFR), or death from kidney-related or cardiovascular causes, by 24% (HR 0.76, 95% CI 0.66–0.88; *p* = 0.0003) [41]. Semaglutide also reduced the risk of cardiovascular death (HR 0.71, 95% CI 0.56–0.89), MACE (HR 0.82, 95% CI 0.68–0.98; *p* = 0.029), and all-cause mortality (HR 0.80, 95% CI 0.67–0.95; *p* = 0.01) [41] while significantly slowing annual eGFR decline. These find-ings established, for the first time, that GLP-1 receptor agonists can improve hard kidney outcomes in addition to reducing atherosclerotic cardiovascular risk. The principal clini-cal trials evaluating GLP-1 receptor agonists and their impact on cardiovascular and renal outcomes across the cardiorenal continuum are summarized in Table 4.

Current evidence supports a complementary role for GLP-1 receptor agonists and SGLT2 inhibitors within the cardiorenal therapeutic framework. While SGLT2 inhibitors remain the cornerstone for preventing heart failure and slowing CKD progression, GLP-1 receptor agonists provide robust protection against atherosclerotic cardiovascular events and, as demonstrated by FLOW, contribute meaningful nephroprotection. Their combined use therefore represents a rational strategy for comprehensive cardiorenal risk reduction in patients with T2DM and chronic kidney disease.

## 5. Finerenone and Cardiorenal Protection

The clinical development of finerenone was preceded by the ARTS and ARTS-DN phase II studies, which demonstrated significant reductions in albuminuria and established the safety profile of nonsteroidal mineralocorticoid receptor antagonism, thereby providing the rationale for the subsequent FIDELIO-DKD and FIGARO-DKD outcome trials. Finerenone, a selective nonsteroidal mineralocorticoid receptor antagonist (ns-MRA), represents a novel therapeutic approach targeting inflammation and fibrosis across the cardiorenal continuum. Unlike steroidal mineralocorticoid receptor antagonists, finerenone exhibits greater receptor selectivity and a lower incidence of off-target adverse effects while preserving potent anti-inflammatory and anti-fibrotic properties. These mechanisms are particularly relevant in type 2 diabetes mellitus (T2DM) and chronic kidney disease (CKD), conditions characterized by persistent mineralocorticoid receptor overactivation that contributes to progressive renal injury, myocardial remodeling, and cardiovascular events. The first evidence of clinically meaningful cardiorenal protection emerged from the FIDELIO-DKD trial, which enrolled patients with T2DM and advanced CKD receiving optimized renin–angiotensin system blockade. Finerenone significantly reduced the risk of kidney failure, with sustained decline in estimated glomerular filtration rate (eGFR) or renal death by 18% (HR 0.82, 95% CI 0.73–0.93; *p* = 0.001) [44], while also reducing the risk of cardiovascular events. These findings established finerenone as the first nonsteroidal mineralocorticoid receptor antagonist capable of improving hard renal outcomes in diabetic kidney disease. Subsequently, FIGARO-DKD expanded the evidence base to a broader CKD population with earlier stages of kidney disease. In that trial, finerenone significantly reduced the composite of cardiovascular death, nonfatal myocardial infarction, nonfatal stroke, or hospitalization for heart failure (HR 0.87, 95% CI 0.76–0.98; *p* = 0.03) [45], with the greatest contribution arising from a reduction in heart failure hospitalizations. Although renal outcomes were directionally favorable, the study was primarily designed to evaluate cardiovascular benefit, thereby highlighting the complementary cardiovascular effects of mineralocorticoid receptor blockade in patients with diabetic kidney disease. The pooled FIDELITY analysis, including more than 13,000 participants from FIDELIO-DKD and FIGARO-DKD, confirmed a consistent reduction in both cardiovascular and renal outcomes, demonstrating a 14% reduction in cardiovascular events and a 23% reduction in kidney outcomes [46]. Importantly, these benefits were observed on top of contemporary guideline-directed medical therapy, supporting finerenone as a foundational component of cardiorenal risk reduction in patients with T2DM and CKD. More recently, the therapeutic spectrum of finerenone has expanded beyond diabetic kidney disease. In the FINEARTS-HF trial, which enrolled patients with heart failure and mildly reduced or preserved ejection fraction (LVEF ≥ 40%), finerenone significantly reduced the composite of total worsening heart failure events and cardiovascular death compared with placebo (rate ratio 0.84, 95% CI 0.74–0.95; *p* = 0.007) [47]. This benefit was primarily driven by a reduction in recurrent worsening heart failure events (rate ratio 0.82, 95% CI 0.71–0.94; *p* = 0.006), whereas cardiovascular mortality was not significantly reduced (HR 0.93, 95% CI 0.78–1.11) [47]. These findings provide the first robust evidence that nonsteroidal mineralocorticoid receptor antagonism can improve outcomes across the spectrum of heart failure beyond reduced ejection fraction, extending the role of finerenone from nephroprotection to broader cardiovascular disease management. Recent evidence has further strengthened the rationale for combination therapy across the cardiorenal–metabolic continuum. In the CONFIDENCE trial, the simultaneous initiation of finerenone and empagliflozin in patients with T2D and CKD resulted in a 29% greater reduction in the urinary albumin-to-creatinine ratio compared with finerenone alone and a 32% greater reduction compared with empagliflozin alone (both *p* < 0.001) [48] without unexpected safety concerns. These findings provide the first randomized evidence supporting the additive cardiorenal effects of combined nonsteroidal mineralocorticoid receptor antagonism and SGLT2 inhibition and may represent an important step toward a more comprehensive cardiorenal–metabolic treatment strategy. The principal clinical trials evaluating finerenone and its role in reducing cardiovascular events and slowing CKD progression across the cardiorenal continuum are summarized in Table 5.

The available literature positions finerenone as a key component of contemporary cardiorenal therapy. Whereas SGLT2 inhibitors primarily target glomerular hemodynamics and heart failure pathways and GLP-1 receptor agonists predominantly reduce atherosclerotic cardiovascular risk, finerenone acts through anti-inflammatory and anti-fibrotic mechanisms that directly address structural organ damage. The complementary biological actions and clinical benefits of these three therapeutic classes support their combined use as the foundation of an integrated cardiorenal–metabolic strategy in patients with T2DM, CKD, and heart failure.

## 6. Albuminuria: From Biomarker to Therapeutic Target

While albuminuria is widely recognized as a strong risk marker and a valuable indicator of treatment response, it is not a universally validated surrogate endpoint for cardiovascular benefit. For instance, with certain pharmacological classes such as SGLT2 inhibitors, significant cardiovascular benefits, including the reduction of hospitalizations for heart failure, are observed even in patients with normoalbuminuria or in the absence of significant changes in albuminuria levels during treatment. The progressive incorporation of albuminuria into clinical trial design and guideline recommendations reflects a broader shift in cardiorenal medicine. Because reductions in albuminuria have consistently predicted improved long-term renal and cardiovascular outcomes across multiple therapeutic classes, albuminuria may represent the closest currently available biomarker to a unifying therapeutic target within the cardiorenal–metabolic continuum.

### 6.1. Hyperkalemia and Renal Function Changes During Cardiorenal–Metabolic Therapy: From Safety Concerns to Therapeutic Opportunities

The remarkable benefits of renin–angiotensin–aldosterone system inhibitors (RAASi), angiotensin receptor–neprilysin inhibitors (ARNIs), SGLT2 inhibitors, and nonsteroidal mineralocorticoid receptor antagonists have transformed the management of patients with heart failure, chronic kidney disease (CKD), diabetes, and cardiovascular disease. However, the use of these therapies is frequently limited by concerns regarding worsening renal function and hyperkalemia. Historically, increases in serum creatinine and potassium levels have often prompted dose reduction or discontinuation of disease-modifying therapies, potentially depriving patients of substantial cardiovascular and renal benefits. Contemporary evidence suggests that many of these changes represent predictable pharmacodynamic effects rather than true toxicity and should therefore be interpreted within the context of the underlying cardiorenal physiology.

### 6.2. Distinguishing Functional eGFR Decline from True Kidney Injury

A central concept in contemporary cardiorenal medicine is the distinction between a hemodynamically mediated decline in estimated glomerular filtration rate (eGFR) and genuine structural kidney injury. RAAS inhibitors, ARNIs, SGLT2 inhibitors, and finere-none all reduce intraglomerular pressure through different mechanisms. ACE inhibitors and ARBs dilate the efferent arteriole, whereas SGLT2 inhibitors restore tubuloglomerular feedback and induce afferent arteriolar vasoconstriction. The net effect is a reduction in glomerular hyperfiltration and a modest early decline in eGFR, typically occurring within the first weeks of treatment. This phenomenon has been consistently observed across ma-jor cardiorenal outcome trials, including CREDENCE [23], DAPA-CKD [24], EM-PA-KIDNEY [25], FIDELIO-DKD [44], and FIGARO-DKD [45]. Importantly, patients with an initial eGFR decline generally had a slower long-term rate of kidney function loss and improved cardiovascular outcomes than placebo-treated individuals. The initial decline in eGFR should be interpreted as an expected hemodynamic adaptation or a tolerable physi-ological effect secondary to changes in intraglomerular pressure induced by medications such as RAASi and SGLT2i rather than a direct marker of therapeutic efficacy.

Current expert consensus generally considers an increase in serum creatinine of up to 30% from baseline after initiation of RAAS inhibition as acceptable, provided that kidney function subsequently stabilizes and there is no evidence of volume depletion, hypotension, severe hyperkalemia, or other causes of acute kidney injury. Conversely, larger or progressive declines in kidney function should prompt evaluation for renal artery stenosis, excessive diuresis, dehydration, nephrotoxic medications, or intrinsic kidney disease. The challenge for clinicians is therefore not to react reflexively to changes in laboratory values but rather to distinguish physiological adaptation from clinically relevant renal injury.

### 6.3. Hyperkalemia: The Major Barrier to Guideline-Directed Medical Therapy

Hyperkalemia remains one of the most important obstacles to the implementation and maintenance of guideline-directed medical therapy. This issue is particularly relevant in patients with CKD, diabetes, and heart failure, where impaired potassium excretion fre-quently coexists with the need for intensive neurohormonal blockade. The paradox of car-diorenal therapy is that the treatments associated with the greatest prognostic bene-fit—including ACE inhibitors, ARBs, ARNIs, steroidal mineralocorticoid receptor antago-nists, and finerenone—are also those most likely to increase serum potassium levels. Data from RALES [51], EPHESUS [52], EMPHASIS-HF [52], FIDELIO-DKD [44], and FINE-ARTS-HF [47] consistently demonstrate that hyperkalemia is more frequent among treated patients. Nevertheless, these same studies showed substantial reductions in mortality, heart failure hospitalization, and CKD progression. Consequently, contemporary guide-lines emphasize that discontinuation of life-saving therapies should be considered only after reversible causes of hyperkalemia have been addressed and alternative management strategies have been explored.

To optimize patient safety and align with recent international guidelines, including KDI-GO 2024 [53] and the European Society of Cardiology on CKD and CVD 2026 [54], the fol-lowing practical framework for monitoring and therapy reassessment is recommended.

Serum creatinine and potassium levels should be systematically evaluated 2 to 4 weeks after the initiation or dose titration of RAAS inhibitors, SGLT2 inhibitors, or MRAs.

An initial increase in serum creatinine of up to 30% from baseline is considered acceptable and should not prompt immediate drug withdrawal, provided that kidney function subsequently stabilizes. However, before attributing an eGFR drop exclusively to the hemodynamic effects of nephroprotective therapies, clinicians must assess and correct for potential confounding factors such as volume depletion, dehydration, or excessive concurrent diuretic use.

Treatment should be temporarily withheld in the event of an acute eGFR decline exceeding 30% from baseline. Furthermore, while the risk of hyperkalemia is notably higher with therapies like finerenone and other MRAs, these agents must be interrupted if severe hyperkalemia (>6.0 mmol/L) occurs and remains unresponsive to corrective measures, including the introduction of novel potassium binders.

### 6.4. New Potassium Binders and the Concept of Enablement Therapy

The development of new potassium binders has changed the management of chronic hyperkalemia from a reactive safety strategy to a proactive tool for maintaining disease-modifying cardiorenal therapies. Hyperkalemia is particularly frequent in patients with chronic kidney disease, diabetes, and heart failure, the same populations in whom RAAS inhibitors, ARNIs, and mineralocorticoid receptor antagonists provide the greatest prognostic benefit. Therefore, potassium binders should not be viewed only as potassium-lowering agents but also as “enablement therapies” that may allow initiation, continuation, and optimization of guideline-directed medical therapy.

Patiromer is a nonabsorbed potassium-binding polymer that exchanges potassium for calcium in the gastrointestinal tract, thereby avoiding sodium loading. In OPAL-HK, patients with chronic kidney disease receiving RAAS inhibitors and presenting with hyperkalemia were treated with patiromer for 4 weeks. Serum potassium decreased by −1.01 ± 0.03 mmol/L from baseline (*p* < 0.001), and 76% of patients achieved the target potassium range. During the randomized withdrawal phase, recurrence of hyperkalemia, defined as serum potassium ≥ 5.5 mmol/L, occurred in 60% of patients switched to placebo compared with 15% of those continuing patiromer (*p* < 0.001) [55]. This trial established the efficacy of patiromer for potassium control in CKD patients treated with RAAS inhibition, but it was not designed to test clinical outcomes or formal RAASi optimization. In AMETHYST-DN, patiromer provided sustained potassium reduction for up to 52 weeks in patients with type 2 diabetes and CKD receiving ACE inhibitors, ARBs, or both, supporting the feasibility of long-term potassium control in a population at high cardiorenal risk [56].

The enablement concept was more directly tested in AMBER, which enrolled patients with resistant hypertension and advanced CKD in whom spironolactone use is often lim-ited by hyperkalemia. Patiromer enabled more persistent spironolactone therapy com-pared with placebo. In the subgroup with eGFR 25 to <30 mL/min/1.73 m^2^, persistent spi-ronolactone use was achieved in 84% of patients receiving patiromer versus 56% receiving placebo (absolute difference 29%; *p* < 0.02) [57]. In the eGFR 30–45 mL/min/1.73 m^2^ sub-group, persistent spironolactone use occurred in 86% versus 69%, respectively (absolute difference 17%; *p* = 0.003) [57], with no significant interaction between eGFR subgroups. These findings demonstrate that patiromer can facilitate mineralocorticoid receptor an-tagonist therapy even in advanced CKD, although the trial was focused on treatment per-sistence and blood pressure reduction rather than hard cardiovascular or renal outcomes.

The DIAMOND trial extended this strategy to patients with heart failure with re-duced ejection fraction and current or previous RAASi-related hyperkalemia. After a run-in period designed to optimize RAASi and MRA therapy while using patiromer, 878 patients were randomized to continue patiromer or switch to placebo. The primary end-point, the between-group difference in adjusted mean serum potassium change, favored patiromer: +0.03 mmol/L in the patiromer group versus +0.13 mmol/L in the placebo group, with a between-group difference of −0.10 mmol/L (95% CI −0.13 to −0.07; *p* < 0.001). Pati-romer also reduced the risk of hyperkalemia events > 5.5 mmol/L (HR 0.63, 95% CI 0.45–0.87; *p* = 0.006), reduced MRA dose reduction (HR 0.62, 95% CI 0.45–0.87; *p* = 0.006), and reduced the total number of hyperkalemia events (HR 0.66, 95% CI 0.53–0.81; *p* < 0.001) [58]. Hyperkalemia-related outcomes also favored patiromer based on win ratio analysis (win ratio 1.53, 95% CI 1.23–1.91; *p* < 0.001). However, the trial was modified during the COVID-19 pandemic from an outcomes-driven design to a potassium control study, and therefore it cannot establish that potassium-binder-enabled RAASi optimization translates into fewer deaths or heart failure hospitalizations. Importantly, MRA discontinuation alone was numerically lower with patiromer but not statistically significant in the main analysis (4.6% vs. 7.1%; HR 0.64, 95% CI 0.36–1.12) [58], underscoring that the clinical value of chronic binder therapy should be individualized.

Sodium zirconium cyclosilicate is a nonabsorbed inorganic cation exchanger that se-lectively traps potassium in exchange for sodium and hydrogen ions and has a more rapid onset of action than patiromer. In HARMONIZE, ambulatory patients with hyperkalemia received open-label sodium zirconium cyclosilicate at 10 g three times daily for 48 h; among those achieving normokalemia, randomized maintenance therapy with 5, 10, or 15 g daily maintained lower serum potassium levels than placebo for 28 days. Normokalemia was achieved in 84% of patients within 24 h and 98% within 48 h, and all three mainte-nance doses resulted in significantly higher proportions of patients with normal potassi-um levels compared with placebo. A pooled analysis of ZS-003 and HARMONIZE among patients with baseline potassium ≥ 5.5 mmol/L confirmed the rapid potassium-lowering effect: 37.5% achieved potassium ≤ 5.0 mmol/L by 4 h and 85.0% by 48 h; median time to potassium ≤ 5.5 mmol/L was 2.0 h (95% CI 1.1–2.0), and median time to potassium ≤ 5.0 mmol/L was 21.6 h (95% CI 4.1–22.4) [59].

The potential role of sodium zirconium cyclosilicate as an enablement therapy in heart failure was evaluated in PRIORITIZE-HF, a randomized, double-blind trial testing whether sodium zirconium cyclosilicate could facilitate RAASi intensification in symp-tomatic HFrEF. The trial was prematurely terminated because of the COVID-19 pandemic after 182 patients had been randomized. At 3 months, there was no statistically significant difference in RAASi treatment categories between sodium zirconium cyclosilicate and placebo (*p* = 0.43), although the proportion of patients reaching the target MRA dose was numerically higher with sodium zirconium cyclosilicate than with placebo (56.4% vs. 47.0%) [60]. Mean potassium levels were lower during treatment, and patient-reported outcomes numerically favored sodium zirconium cyclosilicate, but the study was under-powered and cannot support definitive conclusions regarding RAASi enablement.

At the same time, in the Sodium Zirconium Cyclosilicate for Management of Hyper-kalemia During Spironolactone Optimization in Patients With Heart Failure (REALIZE-K) [61], in 203 participants with HFrEF and hyperkalemia, SZC led to large improvements in the percentage of participants with normokalemia while on an optimal spironolactone dose (OR: 4.45; 95% CI: 2.89–6.86; *p* < 0.001) and reduced risk of hyperkalemia and down-titration/discontinuation of spironolactone (HR: 0.37; 95% CI: 0.17–0.73; *p* = 0.006). Although underpowered for clinical outcomes, more participants had HF events with SZC than placebo, which should be factored into clinical decision making.

The main clinical trials evaluating novel potassium binders and their role in facilitating the implementation and maintenance of guideline-directed cardiorenal therapies are summarized in Table 6.

Taken together, these trials support the efficacy of patiromer and sodium zirconium cyclosilicate for potassium lowering and prevention of recurrent hyperkalemia. Patiromer currently has the strongest evidence for enabling continuation of MRA or RAASi therapy, particularly from AMBER and DIAMOND, whereas sodium zirconium cyclosilicate offers rapid potassium reduction and effective short-term maintenance, with still limited and underpowered evidence for RAASi optimization in heart failure. The clinical implication is that hyperkalemia should no longer automatically lead to withdrawal of life-saving cardiorenal therapies. Instead, potassium binders may be used selectively to maintain RAASi, ARNI, MRA, and finerenone therapy in patients with recurrent or treatment-limiting hyperkalemia. Nevertheless, available studies primarily demonstrate biochemical control and treatment persistence; whether systematic binder-enabled optimization improves hard cardiovascular and renal outcomes remains an important unanswered question.

## 7. Conclusions and Future Perspectives

Over the last four decades, the management of cardiovascular, renal, and metabolic disease has evolved from an organ-specific approach to an integrated cardiorenal–metabolic paradigm. Landmark studies of RAAS inhibitors first established the foundations of cardiorenal protection, while subsequent advances with ARNIs, SGLT2 inhibitors, GLP-1 receptor agonists, and finerenone have expanded this concept by targeting complementary mechanisms involved in disease progression. Current evidence supports the existence of a unified cardiorenal–metabolic continuum in which cardiac dysfunction, chronic kidney disease, diabetes, obesity, inflammation, and vascular disease interact bidirectionally and accelerate one another. Within this framework, contemporary therapies can be viewed according to three major mechanistic axes: the hemodynamic axis, targeted by RAAS inhibitors, ARNIs, and SGLT2 inhibitors; the metabolic and atherosclerotic axis, predominantly modulated by GLP-1 receptor agonists; and the inflammatory–fibrotic axis, targeted by finerenone. This conceptual framework is increasingly endorsed by contemporary cardiovascular, nephrology, and diabetes guidelines, which recognize cardiorenal–metabolic syndrome as a unified disease continuum and advocate for the integrated use of therapies targeting complementary hemodynamic, metabolic, and inflammatory–fibrotic pathways. Such an approach reflects the growing shift from organ-specific treatment strategies to comprehensive risk modification across the entire cardiorenal–metabolic spectrum [53,64,65,66]. The evolution of these therapies has also reshaped the interpretation of traditional renal biomarkers. Albuminuria has emerged as a modifiable therapeutic target and a surrogate marker of residual cardiorenal risk. Likewise, the nephroprotective benefits of SGLT2 inhibitors are observed despite an initial decline in eGFR, a predictable hemodynamic response that should not be mistaken for structural kidney injury. These concepts highlight the importance of avoiding premature discontinuation of therapies with proven long-term cardiorenal benefits. A major challenge in contemporary practice is the implementation and maintenance of optimal multidrug therapy. Increasing evidence supports an integrated strategy combining RAAS inhibition or ARNI therapy, SGLT2 inhibitors, GLP-1 receptor agonists, and finerenone, a paradigm that may be regarded as a form of “quadruple cardiorenal therapy.” It is needed to distinguish the proposed “quadruple cardiorenal therapy” from the established foundational therapy for HFrEF. While the classical HFrEF regimen primarily focuses on specific neurohormonal modulation to preserve cardiac function and reduce mortality, the quadruple cardiorenal approach, encompassing RAAS inhibitors or ARNI, SGLT2 inhibitors, GLP-1 RAs, and finerenone, targets the broader cardiorenal–metabolic continuum. This multidimensional paradigm acts simultaneously on three complementary pathophysiological axes: the hemodynamic axis (optimized by RAASi/ARNI and SGLT2i), the metabolic and atherosclerotic axis (predominantly modulated by GLP-1 RAs), and the inflammatory–fibrotic axis (specifically targeted by finerenone). The use of potassium binders as enablement therapies may further facilitate the continuation of guideline-directed treatment in patients at risk of hyperkalemia. Recent studies have continued to expand the therapeutic horizon. The SELECT trial demonstrated that semaglutide reduces major adverse cardiovascular events in overweight or obese individuals without diabetes, extending the cardiovascular benefits of GLP-1 receptor agonists beyond glycemic control. The CONFIDENCE trial demonstrated greater reductions in albuminuria at 180 days with finerenone and SGLT2 inhibitors but no additional protection against clinical cardiovascular or kidney events. Future research will likely move beyond individual drug classes toward personalized and multimarker-guided treatment strategies. Ultimately, the goal of cardiorenal–metabolic medicine is no longer simply to treat individual organs but to intervene across the entire disease continuum through integrated, mechanism-based, and patient-centered therapeutic approaches.

## Figures and Tables

**Table 1:** Landmark trials of ACE inhibitors and ARBs in cardiorenal protection.

Trial (Drug)	Drug	Patients	Study Population	Cardiovascular Outcomes	Renal Outcomes
CONSENSUS (1987) [9]	Enalapril vs. placebo	*N* = 253 73% male Mean age 70 y	Severe HFrEF (NYHA IV)	6-month mortality: ↓ 40% (RRR; *p* = 0.002) Overall mortality: ↓ 27% (*p* = 0.003)	No prespecified renal outcome
SOLVD-Treatment (1991) [3]	Enalapril vs. placebo	*N* = 2569 80% male Mean age 61 y	HFrEF (LVEF ≤ 35%)	All-cause mortality: RR 0.84 (95% CI 0.74–0.95); *p* = 0.0036 HF hospitalization: ↓ 26%; *p* < 0.0001	No prespecified renal outcome
SAVE (1992) [4]	Captopril vs. placebo	*N* = 2231 82% male Mean age 59 y	Post-MI with asymptomatic LV dysfunction	All-cause mortality: RR 0.81 (95% CI 0.68–0.97); *p* = 0.019 HF hospitalization: ↓ 22%; *p* = 0.019	No prespecified renal outcome
AIRE (1993) [6]	Ramipril vs. placebo	*N* = 2006 74% male Mean age 65 y	Clinical HF after MI	All-cause mortality: RR 0.73 (95% CI 0.60–0.88); *p* = 0.002	No prespecified renal outcome
TRACE (1995) [5]	Trandolapril vs. placebo	*N* = 1749 72% male Mean age 67 y	Post-MI with LVEF ≤ 35%	All-cause mortality: RR 0.78 (95% CI 0.67–0.91); *p* < 0.001 CV mortality: RR 0.75 (95% CI 0.63–0.89); *p* < 0.001	No prespecified renal outcome
HOPE (2000) [7]	Ramipril vs. placebo	*N* = 9297 73% male Mean age 66 y	High-risk vascular disease and/or diabetes without HF	CV death, MI, or stroke: RR 0.78 (95% CI 0.70–0.86); *p* < 0.001 CV death: RR 0.74	Exploratory renal benefit with reduced overt nephropathy in diabetic patients
EUROPA (2003) [8]	Perindopril vs. placebo	*N* = 12,218 85% male Mean age 60 y	Stable CAD without HF	CV death, MI, or cardiac arrest: 20% RRR (95% CI 9–29%); *p* = 0.0003	No prespecified renal outcome
Lewis et al. (1993) [10]	Captopril vs. placebo	*N* = 409 57% male Mean age 35 y	T1D with overt nephropathy	No dedicated CV endpoint	Doubling serum creatinine: ↓ 48%; *p* = 0.007 Death/dialysis/transplant: ↓ 50%; *p* = 0.006
REIN (1997/1999) [11]	Ramipril vs. conventional antihypertensive therapy (non-ACEi)	*N* = 352 80% male Mean age 45–50 y	Proteinuric non-diabetic CKD	No dedicated CV endpoint	ESRD: significantly increased (RR 1.86; 95% CI 1.07–3.26); *p* = 0.027 Marked slowing of GFR decline
IRMA-2 (2001) [12]	Irbesartan vs. placebo	*N* = 590 67% male Mean age 58 y	T2D, hypertension and microalbuminuria	No dedicated CV endpoint	Progression to overt nephropathy:150 mg: HR 0.56 (95% CI 0.31–0.99)300 mg: HR 0.32 (95% CI 0.15–0.65)
RENAAL (2001) [13]	Losartan vs. placebo (plus conventional therapy)	*N* = 1513 63% male Mean age 60 y	T2D with overt nephropathy	HF hospitalization: ↓ 32%; *p* = 0.005	Doubling of serum creatinine, ESKD, or death: ↓ 16%; *p* = 0.02 ESRD: ↓ 28%; *p* = 0.002
IDNT (2001) [14]	Irbesartan vs. amlodipine or placebo	*N* = 1715 66% male Mean age 59 y	T2D with overt nephropathy	No significant CV benefit	Doubling of serum creatinine, ESKD, or death: ↓ 20% vs. placebo; *p* = 0.02 Doubling serum creatinine: ↓ 33% vs. placebo; *p* = 0.003

This table summarizes the landmark randomized clinical trials that established ACE inhibitors and angiotensin receptor blockers as the foundation of contemporary cardiorenal protection. Early studies demonstrated reductions in mortality and heart failure progression, whereas subsequent nephrology trials established RAAS inhibition as the first strategy capable of reducing albuminuria, slowing kidney function decline, and delaying progression to end-stage kidney disease. ACE: angiotensin-converting enzyme; AIRE: Acute Infarction Ramipril Efficacy Study; ARB: angiotensin receptor blocker; CAD: coronary artery disease; CI: confidence interval; CKD: chronic kidney disease; CONSENSUS: Cooperative North Scandinavian Enalapril Survival Study; CV: cardiovascular; eGFR: estimated glomerular filtration rate; ESRD: end-stage kidney disease; EUROPA: European Trial on Reduction of Cardiac Events with Perindopril in Stable Coronary Artery Disease; GFR: glomerular filtration rate; HF: heart failure; HFrEF: heart failure with reduced ejection fraction; HOPE: Heart Outcomes Prevention Evaluation; HR: hazard ratio; IRMA-2: Irbesartan in Patients with Type 2 Diabetes and Microalbuminuria 2; LVEF: left ventricular ejection fraction; MI: myocardial infarction; NYHA: New York Heart Association; RAAS: renin–angiotensin–aldosterone system; REIN: Ramipril Efficacy in Nephropathy; RENAAL: Reduction of Endpoints in NIDDM; RR: relative risk; RRR: relative risk reduction; SAVE: Survival and Ventricular Enlargement Trial; SOLVD: Studies of Left Ventricular Dysfunction; T1D: type 1 diabetes; T2D: type 2 diabetes; TRACE: Trandolapril Cardiac Evaluation Study.

**Table 2:** Landmark trials of angiotensin receptor–neprilysin inhibition across the cardiorenal continuum.

Trial	Drug	Patients	Study Population	Cardiovascular Outcomes	Renal Outcomes
PARADIGM-HF (2014) [18]	Sacubitril/valsartan vs. enalapril	*N* = 8442 78% male Mean age 64 y	HFrEF (LVEF ≤ 40%)	CV death or first HF hospitalization: HR 0.80 (95% CI 0.73–0.87); *p* < 0.001 All-cause mortality: HR 0.84 (95% CI 0.76–0.93)	Annual eGFR decline: −1.61 vs. −2.04 mL/min/1.73 m^2^/year vs. enalapril (*p* < 0.001), despite a modest increase in albuminuria
PARAGON-HF (2019) [15]	Sacubitril/valsartan vs. valsartan	*N* = 4822 52% male Mean age 73 y	HFpEF (LVEF ≥ 45%)	Total HF hospitalizations and CV death: RR 0.87 (95% CI 0.75–1.01); *p* = 0.06	Annual eGFR decline: −1.8 vs. −2.4 mL/min/1.73 m^2^/year vs. valsartan ≥ 50% decline in eGFR, ESKD, or renal death: HR 0.50 (95% CI 0.33–0.77)
PARADISE-MI (2021) [16]	Sacubitril/valsartan vs. ramipril	*N* = 5661 76% male Mean age 64 y	Acute MI with LV dysfunction and/or pulmonary congestion	CV death, HF hospitalization, or outpatient HF: HR 0.90 (95% CI 0.78–1.04); *p* = 0.17	No prespecified renal outcome
PARAGLIDE-HF (2023) [17]	Sacubitril/valsartan vs. valsartan	*N* = 466 57% male Mean age 70 y	Recent worsening HFmrEF/HFpEF	Time-averaged proportional change in NT-proBNP: Ratio of change 0.85 (95% CI 0.73–0.999); *p* = 0.049	No excess deterioration in kidney function

This table summarizes the landmark randomized clinical trials that established angiotensin receptor–neprilysin inhibition as the evolution of neurohormonal modulation in heart failure. ARNIs have consistently slowed kidney function decline while preserving long-term renal function, supporting their role as a cornerstone of contemporary cardiorenal–metabolic therapy. ARNI: angiotensin receptor–neprilysin inhibitor; CI: confidence interval; CV: cardiovascular; eGFR: estimated glomerular filtration rate; HF: heart failure; HFmrEF: heart failure with mildly reduced ejection fraction; HFpEF: heart failure with preserved ejection fraction; HFrEF: heart failure with reduced ejection fraction; HR: hazard ratio; LVEF: left ventricular ejection fraction; MI: myocardial infarction; NT-proBNP: N-terminal pro-B-type natriuretic peptide; PARADISE-MI: Prospective ARNI versus ACE Inhibitor Trial to Determine Superiority in Reducing Heart Failure Events After Myocardial Infarction; PARADIGM-HF: Prospective Comparison of ARNI with ACEI to Determine Impact on Global Mortality and Morbidity in Heart Failure; PARAGLIDE-HF: Prospective Comparison of ARNI with ARB Given Following Stabilization in Decompensated HF with Preserved Ejection Fraction; PARAGON-HF: Prospective Comparison of ARNI with ARB Global Outcomes in HF with Preserved Ejection Fraction; RAAS: renin–angiotensin–aldosterone system.

**Table 3:** Landmark SGLT2 inhibitor trials across the cardiorenal continuum.

Trial	Drug	Patients	Study Population	Cardiovascular Outcomes	Renal Outcomes
EMPA-REG OUTCOME (2015) [19]	Empagliflozin vs. placebo	*N* = 7020 72% male Mean age 63 y	T2D + established ASCVD	3-point MACE: HR 0.86 (95% CI 0.74–0.99); *p* = 0.04 CV death: HR 0.62 (95% CI 0.49–0.77); *p* < 0.001	Incident or worsening nephropathy: HR 0.61 (95% CI 0.53–0.70); *p* < 0.001
CANVAS Program (2017) [20]	Canagliflozin vs. placebo	*N* = 10,142 64% male Mean age 63 y	T2D + high CV risk	3-point MACE: HR 0.86 (95% CI 0.75–0.97); *p* = 0.02	Sustained 40% eGFR decline, RRT, or renal death: HR 0.60 (95% CI 0.47–0.77) (nominal significance *)
DECLARE–TIMI 58 (2018) [21]	Dapagliflozin vs. placebo	*N* = 17,160 63% male Mean age 64 y	T2D with or at risk for ASCVD	CV death or HF hospitalization: HR 0.83 (95% CI 0.73–0.95); *p* = 0.005	Sustained ≥40% decrease in eGFR to <60 mL/min/1.73 m^2^, new ESKD, or renal/CV death: HR 0.76 (95% CI 0.67–0.87); *p* < 0.001
DAPA-HF (2019) [27]	Dapagliflozin vs. placebo	*N* = 4744 77% male Mean age 66 y	HFrEF (LVEF ≤ 40%), with or without diabetes	Worsening HF or CV death: HR 0.74 (95% CI 0.65–0.85); *p* < 0.001	Annual eGFR decline: −1.09 vs. −2.85 mL/min/1.73 m^2^/year: *p* < 0.001
CREDENCE (2019) [23]	Canagliflozin vs. placebo	*N* = 4401 66% male Mean age 63 y	T2D + albuminuric CKD	HF hospitalization: HR 0.61 (95% CI 0.47–0.80); *p* < 0.001	ESKD, doubling of serum creatinine, or renal/CV death: HR 0.70 (95% CI 0.59–0.82); *p* < 0.001 ESKD, doubling of serum creatinine, or renal death: HR 0.66 (95% CI 0.53–0.81); *p* < 0.001
EMPEROR-Reduced (2020) [32]	Empagliflozin vs. placebo	*N* = 3730 76% male Mean age 67 y	HFrEF (LVEF ≤ 40%), with or without diabetes	CV death or HF hospitalization: HR 0.75 (95% CI 0.65–0.86); *p* < 0.001	Annual eGFR decline: −0.55 vs. −2.28 mL/min/1.73 m^2^/year; *p* < 0.001
VERTIS-CV (2020) [22]	Ertugliflozin vs. placebo	*N* = 8246 70% male Mean age 64 y	T2D + established ASCVD	3-point MACE: HR 0.97 (95% CI 0.85–1.11); *p* < 0.001 for non-inferiority	Renal death, RRT, or doubling of serum creatinine: HR 0.81 (95% CI 0.63–1.04); NS
DAPA-CKD (2020) [24]	Dapagliflozin vs. placebo	*N* = 4304 67% male Mean age 62 y	CKD with or without diabetes	CV death or HF hospitalization: HR 0.71 (95% CI 0.55–0.92); *p* = 0.009	Sustained ≥50% decline in eGFR, ESKD, or renal death: HR 0.56 (95% CI 0.45–0.68); *p* < 0.001.
SOLOIST-WHF (2021) [31]	Sotagliflozin vs. placebo	*N* = 1222 67% male Mean age 70 y	Recent worsening HF + T2D	CV death, HF hospitalization, or urgent HF visit: HR 0.67 (95% CI 0.52–0.85); *p* < 0.001	No excess acute kidney injury
EMPEROR-Preserved (2021) [28]	Empagliflozin vs. placebo	*N* = 5988 55% male Mean age 72 y	HFmrEF/HFpEF (LVEF > 40%)	CV death or HF hospitalization: HR 0.79 (95% CI 0.69–0.90); *p* < 0.001	Annual eGFR decline: −1.25 vs. −2.62 mL/min/1.73 m^2^/year: *p* < 0.001
SCORED (2021) [30]	Sotagliflozin vs. placebo	*N* = 10,584 55% male Mean age 69 y	T2D + CKD	CV death, HF hospitalization, or urgent HF visit: HR 0.74 (95% CI 0.63–0.88); *p* < 0.001	No adequately powered renal outcome (trial terminated early ‡)
DELIVER (2022) [29]	Dapagliflozin vs. placebo	*N* = 6263 56% male Mean age 72 y	HFmrEF/HFpEF (LVEF > 40%)	Worsening HF or CV death: HR 0.82 (95% CI 0.73–0.92); *p* < 0.001	In a prespecified analysis, an initial eGFR decline >10% was not associated with a higher risk of the primary CV endpoint (aHR: 1.62; 95%CI: 0.90–2.89)
EMPA-KIDNEY (2023) [25]	Empagliflozin vs. placebo	*N* = 6609 67% male Mean age 64 y	CKD with or without diabetes	Hospitalization for any cause: HR 0.86 (95% CI 0.78–0.95); *p* = 0.003	Kidney disease progression or CV death: HR 0.72 (95% CI 0.64–0.82); *p* < 0.001

This table summarizes the landmark randomized clinical trials that established SGLT2 inhibitors as disease-modifying therapies across the cardiorenal–metabolic continuum. Their clinical development followed a progressive expansion from cardiovascular outcome trials in patients with T2D to heart failure treatment irrespective of diabetic status and, ultimately, to dedicated nephroprotection in chronic kidney disease. * Renal outcomes in the CANVAS Program were favorable but did not reach formal statistical significance according to the prespecified hierarchical testing strategy. † Composite of renal death, renal replacement therapy, or doubling of serum creatinine. ‡ The SCORED trial was terminated prematurely because of loss of funding, limiting definitive conclusions regarding renal outcomes. ASCVD: atherosclerotic cardiovascular disease; CANVAS: Canagliflozin Cardiovascular Assessment Study; CI: confidence interval; CKD: chronic kidney disease; CREDENCE: Canagliflozin and Renal Events in Diabetes with Established Nephropathy Clinical Evaluation; CV: cardiovascular; CVOT: cardiovascular outcomes trial; DAPA-CKD: Dapagliflozin and Prevention of Adverse Outcomes in Chronic Kidney Disease; DAPA-HF: Dapagliflozin and Prevention of Adverse Outcomes in Heart Failure; DECLARE–TIMI 58: Dapagliflozin Effect on Cardiovascular Events–Thrombolysis in Myocardial Infarction 58; DELIVER: Dapagliflozin Evaluation to Improve the LIVEs of Patients With Preserved Ejection Fraction Heart Failure; eGFR: estimated glomerular filtration rate; EMPA-KIDNEY: The Study of Heart and Kidney Protection With Empagliflozin; EMPA-REG OUTCOME: Empagliflozin Cardiovascular Outcome Event Trial in Type 2 Diabetes Mellitus Patients; EMPEROR-Preserved: Empagliflozin Outcome Trial in Patients with Chronic Heart Failure and Preserved Ejection Fraction; EMPEROR-Reduced: Empagliflozin Outcome Trial in Patients with Chronic Heart Failure and Reduced Ejection Fraction; ESKD: end-stage kidney disease; HF: heart failure; HFmrEF: heart failure with mildly reduced ejection fraction; HFpEF: heart failure with preserved ejection fraction; HFrEF: heart failure with reduced ejection fraction; HR: hazard ratio; MACE: major adverse cardiovascular events; RRT: renal replacement therapy; SCORED: Effect of Sotagliflozin on Cardiovascular and Renal Events in Patients with Type 2 Diabetes and Moderate Renal Impairment Who Are at Cardiovascular Risk; SGLT2: sodium–glucose cotransporter 2; SOLOIST-WHF: Effect of Sotagliflozin on Cardiovascular Events in Patients with Type 2 Diabetes Post Worsening Heart Failure; T2D: type 2 diabetes; VERTIS-CV: Evaluation of Ertugliflozin Efficacy and Safety Cardiovascular Outcomes Trial.

**Table 4:** Landmark GLP-1 receptor agonist trials across the cardiorenal continuum.

Trial	Drug	Patients	Study Population	Cardiovascular Outcomes	Renal Outcomes
ELIXA (2015) [33]	Lixisenatide vs. placebo	*N* = 6068 69% male Mean age 60 y	T2D with recent acute coronary syndrome	Primary composite (CV death, MI, stroke, or unstable angina): HR 1.02 (95% CI 0.89–1.17); *p* = 0.81 Established cardiovascular safety	No prespecified renal outcome
LEADER (2016) [35]	Liraglutide vs. placebo	*N* = 9340 64% male Mean age 64 y	T2D with high cardiovascular risk	3-point MACE: HR 0.87 (95% CI 0.78–0.97); *p* = 0.01 CV death: HR 0.78 (95% CI 0.66–0.93); *p* = 0.007	Nephropathy events: HR 0.78 (95% CI 0.67–0.92); *p* = 0.003
SUSTAIN-6 (2016) [36]	Semaglutide (subcutaneous) vs. placebo	*N* = 3297 61% male Mean age 65 y	T2D with high cardiovascular risk	3-point MACE: HR 0.74 (95% CI 0.58–0.95); *p* = 0.02	New or worsening nephropathy: HR 0.64 (95% CI 0.46–0.88); *p* = 0.005
EXSCEL (2017) [34]	Once-weekly exenatide vs. placebo	*N* = 14,752 62% male Mean age 62 y	T2D with or without established CVD	3-point MACE: HR 0.91 (95% CI 0.83–1.00); *p* = 0.06	No prespecified renal outcome
HARMONY Outcomes (2018) [42]	Albiglutide vs. placebo	*N* = 9463 70% male Mean age 64 y	T2D with established ASCVD	3-point MACE: HR 0.78 (95% CI 0.68–0.90); *p* = 0.0006	No prespecified renal outcome
PIONEER-6 (2019) [43]	Oral semaglutide vs. placebo	*N* = 3183 68% male Mean age 66 y	T2D with high CV risk or CKD	3-point MACE: HR 0.79 (95% CI 0.57–1.11); non-inferiority achieved	No prespecified renal outcome
REWIND (2019) [37]	Dulaglutide vs. placebo	*N* = 9901 54% male Mean age 66 y	T2D with or without previous CVD	3-point MACE: HR 0.88 (95% CI 0.79–0.99); *p* = 0.026	No dedicated renal endpoint. New macroalbuminuria, sustained ≥30% decline in eGFR from baseline, or KRT: HR 0.85 (95% CI 0.77–0.93); *p* = 0.0004
AMPLITUDE-O (2021) [39]	Efpeglenatide vs. placebo	*N* = 4076 67% male Mean age 65 y	T2D with established CVD or CKD plus cardiovascular risk factors	3-point MACE: HR 0.73 (95% CI 0.58–0.92); *p* = 0.007	New macroalbuminuria, ≥40% decline in eGFR for ≥30 days, KRT for ≥90 days, or sustained eGFR <15 mL/min/1.73 m^2^): HR 0.68 (95% CI 0.57–0.79); *p* < 0.001
SELECT (2023) [40]	Semaglutide 2.4 mg vs. placebo	*N* = 17,604 72% male Mean age 62 y	Overweight or obese adults with established ASCVD without diabetes	3-point MACE: HR 0.80 (95% CI 0.72–0.90); *p* < 0.001 All-cause mortality: HR 0.81 (95% CI 0.71–0.93)	In a prespecified analysis, main composite kidney endpoint was lower with semaglutide versus placebo (HR = 0.78; 95% CI 0.63, 0.96; *p* = 0.02)
FLOW (2024) [41]	Semaglutide vs. placebo	*N* = 3533 70% male Mean age 66 y	T2D with CKD	3-point MACE: HR 0.82 (95% CI 0.68–0.98); *p* = 0.029 CV death: HR 0.71 (95% CI 0.56–0.89)	Kidney failure, ≥50% decline in eGFR, or kidney or CV death: HR 0.76 (95% CI 0.66–0.88); *p* = 0.0003 Annual eGFR slope: slower decline by 1.16 mL/min/1.73 m^2^/year (*p* < 0.001)

This table summarizes the landmark randomized clinical trials that established GLP-1 receptor agonists as disease-modifying therapies across the cardiorenal–metabolic continuum. Clinical development progressed from cardiovascular safety studies to trials demonstrating reductions in major adverse cardiovascular events in patients with type 2 diabetes, followed by dedicated evidence of nephroprotection in chronic kidney disease (FLOW) and cardiovascular benefit in overweight and obese individuals without diabetes (SELECT). AMPLITUDE-O: Effect of Efpeglenatide on Cardiovascular Outcomes; ASCVD: atherosclerotic cardiovascular disease; CI: confidence interval; CKD: chronic kidney disease; CV: cardiovascular; eGFR: estimated glomerular filtration rate; ELIXA: Evaluation of Lixisenatide in Acute Coronary Syndrome; EXSCEL: Exenatide Study of Cardiovascular Event Lowering; FLOW: Effect of Semaglutide versus Placebo on the Progression of Renal Impairment in Subjects with Type 2 Diabetes and Chronic Kidney Disease; GLP-1: glucagon-like peptide-1; GLP-1 RA: glucagon-like peptide-1 receptor agonist; HARMONY Outcomes: Albiglutide and Cardiovascular Outcomes in Patients with Type 2 Diabetes and Cardiovascular Disease; HF: heart failure; HR: hazard ratio; KRT: kidney replacement therapy; LEADER: Liraglutide Effect and Action in Diabetes: Evaluation of Cardiovascular Outcome Results; MACE: major adverse cardiovascular events; MI: myocardial infarction; PIONEER-6: Peptide Innovation for Early Diabetes Treatment; REWIND: Researching Cardiovascular Events with a Weekly Incretin in Diabetes; SELECT: Semaglutide Effects on Cardiovascular Outcomes in People With Overweight or Obesity; SUSTAIN-6: Trial to Evaluate Cardiovascular and Other Long-term Outcomes with Semaglutide in Subjects with Type 2 Diabetes; T2D: type 2 diabetes; UACR: urinary albumin-to-creatinine ratio.

**Table 5:** Landmark finerenone trials across the cardiorenal continuum.

Trial	Drug	Patients	Study Population	Cardiovascular Outcomes	Renal Outcomes
ARTS (2013) [49]	Finerenone vs. spironolactone	*N* = 392 75% male Mean age 69 y	HFrEF with CKD	Similar efficacy to spironolactone with a lower incidence of hyperkalemia	No prespecified renal outcome
ARTS-DN (2015) [50]	Finerenone vs. placebo	*N* = 823 78% male Mean age 64 y	T2D with CKD and albuminuria	No dedicated cardiovascular endpoint	UACR reduction: up to 38% vs. placebo: *p* < 0.001
FIDELIO-DKD (2020) [45]	Finerenone vs. placebo	*N* = 5734 70% male Mean age 66 y	T2D with advanced CKD	CV death, MI, stroke, or HF hospitalization: HR 0.86 (95% CI 0.75–0.99); *p* = 0.03	Kidney failure, sustained ≥40% eGFR decline, or renal death: HR 0.82 (95% CI 0.73–0.93); *p* = 0.001
FIGARO-DKD (2021) [44]	Finerenone vs. placebo	*N* = 7437 69% male Mean age 64 y	T2D with earlier-stage CKD	CV death, MI, stroke, or HF hospitalization: HR 0.87 (95% CI 0.76–0.98); *p* = 0.03 HF hospitalization: HR 0.71 (95% CI 0.56–0.90)	Kidney failure, sustained ≥40% eGFR decline, or renal death: HR 0.87 (95% CI 0.76–1.01); *p* = 0.069
FIDELITY (2022) [46]	Finerenone vs. placebo	*N* = 13,171 70% male Mean age 65 y	Pooled analysis of FIDELIO-DKD and FIGARO-DKD	CV death, MI, stroke, or HF hospitalization: HR 0.86 (95% CI 0.78–0.95); *p* < 0.01	Kidney failure, sustained eGFR decline, or renal death: HR 0.77 (95% CI 0.67–0.88); *p* < 0.001
FINEARTS-HF (2024) [47]	Finerenone vs. placebo	*N* = 6001 55% male Mean age 72 y	HFmrEF/HFpEF (LVEF ≥ 40%)	Primary endpoint (total worsening HF events + CV death): Rate ratio 0.84 (95% CI 0.74–0.95); *p* = 0.007 Total worsening HF events: Rate ratio 0.82 (95% CI 0.71–0.94); *p* = 0.006	No prespecified renal outcome
CONFIDENCE (2025) [48]	Finerenone + empagliflozin vs. finerenone alone vs. empagliflozin alone	*N* = 818 69% male Mean age 68 y	T2D with CKD and albuminuria receiving background RAAS inhibition	No dedicated cardiovascular endpoint	UACR reduction ratio 0.71 and 0.68: *p* < 0.001

This table summarizes the landmark randomized clinical trials that established finerenone as the first nonsteroidal mineralocorticoid receptor antagonist with proven cardiorenal benefits. Clinical development progressed from mechanistic studies demonstrating albuminuria reduction and renal safety to large outcome trials showing reductions in kidney disease progression and cardiovascular events in patients with type 2 diabetes and chronic kidney disease. More recently, benefits have been extended to heart failure with mildly reduced or preserved ejection fraction (FINEARTS-HF), while the CONFIDENCE trial provided the first randomized evidence supporting additive albuminuria reduction with combined finerenone and SGLT2 inhibitor therapy. ARTS: Mineralocorticoid Receptor Antagonist Tolerability Study; ARTS-DN: Mineralocorticoid Receptor Antagonist Tolerability Study–Diabetic Nephropathy; CI: confidence interval; CKD: chronic kidney disease; CONFIDENCE: COmbinatioN effect of FInerenone anD EmpaglifloziN in participants with CKD and T2D using a UACR Endpoint; CV: cardiovascular; eGFR: estimated glomerular filtration rate; FIDELIO-DKD: Finerenone in Reducing Kidney Failure and Disease Progression in Diabetic Kidney Disease; FIDELITY: Finerenone in Chronic Kidney Disease and Type 2 Diabetes: Combined FIDELIO-DKD and FIGARO-DKD Analysis; FIGARO-DKD: Finerenone in Reducing Cardiovascular Mortality and Morbidity in Diabetic Kidney Disease; FINEARTS-HF: Finerenone Trial to Investigate Efficacy and Safety Superior to Placebo in Patients with Heart Failure and Preserved Ejection Fraction; HF: heart failure; HFmrEF: heart failure with mildly reduced ejection fraction; HFpEF: heart failure with preserved ejection fraction; HR: hazard ratio; MI: myocardial infarction; MRA: mineralocorticoid receptor antagonist; RAAS: renin–angiotensin–aldosterone system; SGLT2: sodium–glucose cotransporter 2; T2D: type 2 diabetes; UACR: urinary albumin-to-creatinine ratio.

**Table 6:** Landmark trials of novel potassium binders enabling guideline-directed cardiorenal therapy.

Trial	Drug	Patients	Study Population	Primary Efficacy Result	Enablement Outcome
HARMONIZE (2014) [59]	SZC vs. placebo	*N* = 258 61% male Mean age 65 y	Ambulatory hyperkalemia	Normokalemia at day 29: 80%, 90% and 94% vs. 46% with placebo (*p* < 0.001)	Established rapid correction and maintenance of normokalemia
OPAL-HK (2015) [55]	Patiromer vs. placebo *	*N* = 243 58% male Mean age 64 y	CKD + hyperkalemia on RAASi	Serum K^+^: −1.01 ± 0.03 mmol/L (*p* < 0.001); recurrent hyperkalemia 15% vs. 60% (*p* < 0.001)	Enabled continuation of RAAS inhibitor therapy
AMETHYST-DN (2015) [56]	Patiromer (open-label)	*N* = 306 66% male Mean age 66 y	T2D + CKD + hyperkalemia	Mean serum K^+^ maintained within target range through 52 weeks	Demonstrated long-term potassium control during RAASi therapy
ZS-003 (2015) [62]	SZC vs. placebo	*N* = 753 60% male Mean age 66 y	Mixed hyperkalemia	Serum K^+^ reduction: significant within 1 h; dose-dependent reduction at 48 h (*p* < 0.001)	Fastest onset of action among chronic potassium binders
Pooled ZS-003 + HARMONIZE (2019) [63]	SZC	*N* = 170 62% male Mean age 66 y	Moderate-to-severe hyperkalemia	Median time to K^+^ ≤ 5.5 mmol/L:2.0 h; K^+^ ≤ 5.0 mmol/L: 21.6 h	Confirmed rapid potassium normalization
AMBER (2019) [57]	Patiromer vs. placebo	*N* = 295 52% male Mean age 68 y	CKD + resistant hypertension starting spironolactone	Persistent spironolactone use: 86% vs. 69% (*p* = 0.003)	First proof of “enablement therapy” for MRA optimization
DIAMOND (2022) [58]	Patiromer vs. placebo	*N* = 878 75% male Mean age 67 y	HFrEF + current/prior hyperkalemia	Hyperkalemia > 5.5 mmol/L: HR 0.63 (95% CI 0.45–0.87); Δ serum K^+^: −0.10 mmol/L (*p* < 0.001)	Reduced MRA dose reduction/discontinuation and maintained GDMT
PRIORITIZE-HF (2023) [60]	SZC vs. placebo	*N* = 182 74% male Mean age 69 y	HFrEF requiring RAASi optimization	Target MRA dose: 56.4% vs. 47.0% (*p* = 0.43)	Trend toward RAASi optimization; underpowered due to early termination
REALIZE- K (2025) [61]	SCZ vs. placebo	*N* = 202 75% male Mean age 72 y	HFrEF requiring spironolactone optimization	Target MRA dose: 81% vs. 50% (*p* < 0.001)	Trend toward MRA optimization; underpowered for clinical outcomes

This table summarizes the landmark clinical trials evaluating novel potassium binders for hyperkalemia management across the cardiorenal continuum. Clinical development progressed from demonstrating rapid and sustained potassium lowering to establishing the concept of enablement therapy, whereby correction of hyperkalemia facilitates initiation, optimization, and maintenance of guideline-directed cardiorenal therapies, particularly renin–angiotensin–aldosterone system inhibitors and mineralocorticoid receptor antagonists. Trial efficacy is reported as the primary potassium-lowering or treatment enablement outcome, as dedicated cardiovascular or kidney outcome endpoints were not assessed. ARTS: Mineralocorticoid Receptor Antagonist Tolerability Study; ARTS-DN: Mineralocorticoid Receptor Antagonist Tolerability Study–Diabetic Nephropathy; CI: confidence interval; CKD: chronic kidney disease; CONFIDENCE: COmbinatioN effect of FInerenone anD EmpaglifloziN in participants with CKD and T2D using a UACR Endpoint; CV: cardiovascular; eGFR: estimated glomerular filtration rate; FIDELIO-DKD: Finerenone in Reducing Kidney Failure and Disease Progression in Diabetic Kidney Disease; FIDELITY: Finerenone in Chronic Kidney Disease and Type 2 Diabetes: Combined FIDELIO-DKD and FIGARO-DKD Analysis; FIGARO-DKD: Finerenone in Reducing Cardiovascular Mortality and Morbidity in Diabetic Kidney Disease; FINEARTS-HF: Finerenone Trial to Investigate Efficacy and Safety Superior to Placebo in Patients with Heart Failure and Preserved Ejection Fraction; HF: heart failure; HFmrEF: heart failure with mildly reduced ejection fraction; HFpEF: heart failure with preserved ejection fraction; HR: hazard ratio; MI: myocardial infarction; MRA: mineralocorticoid receptor antagonist; RAAS: renin–angiotensin–aldosterone system; REALIZE-K: Sodium Zirconium Cyclosilicate for Management of Hyperkalemia During Spironolactone Optimization in Patients With Heart Failure; SGLT2: sodium–glucose cotransporter 2; SZC: Sodium Zirconium Cyclosilicate; T2D: type 2 diabetes; UACR: urinary albumin-to-creatinine ratio.

## Data Availability

No new data were created or analyzed in this study.
